# Benefit of Introgression Depends on Level of Genetic Trait Variation in Cereal Breeding Programmes

**DOI:** 10.3389/fpls.2022.786452

**Published:** 2022-06-15

**Authors:** Yongjun Li, Fan Shi, Zibei Lin, Hannah Robinson, David Moody, Allan Rattey, Jayfred Godoy, Daniel Mullan, Gabriel Keeble-Gagnere, Matthew J. Hayden, Josquin F. G. Tibbits, Hans D. Daetwyler

**Affiliations:** ^1^Agriculture Victoria, AgriBio, Centre for AgriBioscience, Bundoora, VIC, Australia; ^2^InterGrain, Bibra Lake, WA, Australia; ^3^School of Applied Systems Biology, La Trobe University, Bundoora, VIC, Australia

**Keywords:** introgression, disease resistance, linkage drag, genomic selection, cereal breeding programme

## Abstract

We investigated the benefit from introgression of external lines into a cereal breeding programme and strategies that accelerated introgression of the favourable alleles while minimising linkage drag using stochastic computer simulation. We simulated genomic selection for disease resistance and grain yield in two environments with a high level of genotype-by-environment interaction (G × E) for the latter trait, using genomic data of a historical barley breeding programme as the base generation. Two populations (existing and external) were created from this base population with different allele frequencies for few (*N* = 10) major and many (*N* ~ 990) minor simulated disease quantitative trait loci (QTL). The major disease QTL only existed in the external population and lines from the external population were introgressed into the existing population which had minor disease QTL with low, medium and high allele frequencies. The study revealed that the benefit of introgression depended on the level of genetic variation for the target trait in the existing cereal breeding programme. Introgression of external resources into the existing population was beneficial only when the existing population lacked variation in disease resistance or when minor disease QTL were already at medium or high frequency. When minor disease QTL were at low frequencies, no extra genetic gain was achieved from introgression. More benefit in the disease trait was obtained from the introgression if the major disease QTL had larger effect sizes, more selection emphasis was applied on disease resistance, or more external lines were introgressed. While our strategies to increase introgression of major disease QTL were generally successful, most were not able to completely avoid negative impacts on selection for grain yield with the only exception being when major introgression QTL effects were very large. Breeding programmes are advised to carefully consider the level of genetic variation in a trait available in their breeding programme before deciding to introgress germplasms.

## Introduction

Cereal breeding aims to improve the characteristics of crops to achieve higher grain yield, better quality and more resistance or tolerance to abiotic and biotic stressors so that they become more desirable agronomically and economically. Selection reduces genetic diversity in a population due to a limited number of individuals being chosen as parents of the next generation. Therefore, preserving or broadening the genetic diversity of a population becomes an important task for plant breeders to increase long-term genetic gain. Introgression has been used to transfer single or multiple favourable alleles from wild relatives, landraces or other breeding programmes to broaden genetic diversity or introduce novel traits to an existing breeding programme (Schouten et al., 2019; Hernandez et al., 2020).

Multiple traits usually need to be improved simultaneously in cereal breeding programmes. When introgressing external resources into a cereal breeding programme, the trait of interest usually has a higher performance, but other traits often have lower performance than those in the existing elite breeding programme (Prohens et al., 2017). For example in barley, introgression of Caesarea 26–24 into Harrington contributed some favourable alleles for agronomic and malting traits but also introduced inferior phenotypes of those traits (Matus et al., 2003). Linkage drag is a concern that prevents breeders from utilising introgression into their breeding programmes on a large scale due to a failure of differentiating the rare combinations of chromosomal segments with favourable alleles from inferior alleles (Lukaszewski et al., 2005; Hao et al., 2020).

With the development of genomic tools to facilitate the selection of desirable genotypes, introgression breeding is returning as a mainstream activity (Prohens et al., 2017; Hao et al., 2020). Expected genetic donor contribution was increased in backcrossed lines generated by genomic selection (Odegård et al., 2009a,b). Genomic selection has been widely applied in plant and livestock breeding. It is a method of estimating the genetic potential of selection candidates by using genome-wide polymorphisms and performance records (Meuwissen et al., 2001). It is also a useful strategy for rapid introgression of exotic germplasm in an existing breeding programme (Bernardo, 2009; Odegård et al., 2009a,b). It speeds up the process of introgression of a gene, simultaneously increasing genetic gain and reducing the inbreeding rate (Gaspa et al., 2015).

Computer simulation is an efficient means to model different breeding strategies and predict their long-term performance without a labour-intensive and time-consuming field experiment (Li et al., 2012). Different from the deterministic simulations that are designed to capture some underlying mechanism or a natural process based on equations without random variables and no degree of randomness (Hahl and Kremling, 2016), stochastic simulations are more suitable to mimic an entire plant population under selection for multiple breeding cycles, using a large genomic sequencing datasets generated from the high-throughput molecular biology techniques (Pedersen et al., 2009; Liu et al., 2019). The simulations can provide insight into the identification of best strategies of maximising genetic gain, preserving genetic diversity and optimising operational costs by shortening generation interval, optimising parental crosses or introducing trait variations from external resources. Computer simulations of introgression in plant and livestock breeding have been conducted to identify chromosome segment origin (Amador et al., 2014), to model the incorporation of external genes into another population and quantify changes in gene frequency, inbreeding and genetic gain (Mueller et al., 2018), to improve efficiency of parental selection (Moeinizade et al., 2021) and multi-allelic trait introgression (Han et al., 2017) and to optimise experimental design for optimal trait introgression (Han et al., 2017). However, simulation studies have not investigated genetic architectures that combined many minor loci with effects with few large effect loci originating from outside the breeding programme. We developed stochastic computer simulations of a generic cereal breeding programme for introgression of novel germplasm *via* genomic selection in a commercial cereal breeding programme using barley as an example crop. Barley is widely grown in Australia, with 9 million tonnes of annual production from around 4 million hectares of cultivation, 70% of which is exported, mainly to Asia.^1^ The simulations utilised targeted genotyping-by-sequencing (tGBS) data of commercial barley breeding lines as a starting point and simulated selection over several breeding cycles.

This study investigated the benefit of a one-time introgression of external lines from other germplasm sources into a cereal breeding programme at the beginning of breeding programme. It also examined the strategies that could accelerate the introgression of favourable alleles in disease resistance while minimising linkage drag in other traits (e.g., grain yield).

## Materials and Methods

### Breeding Programme Structure

**Figure 1:**
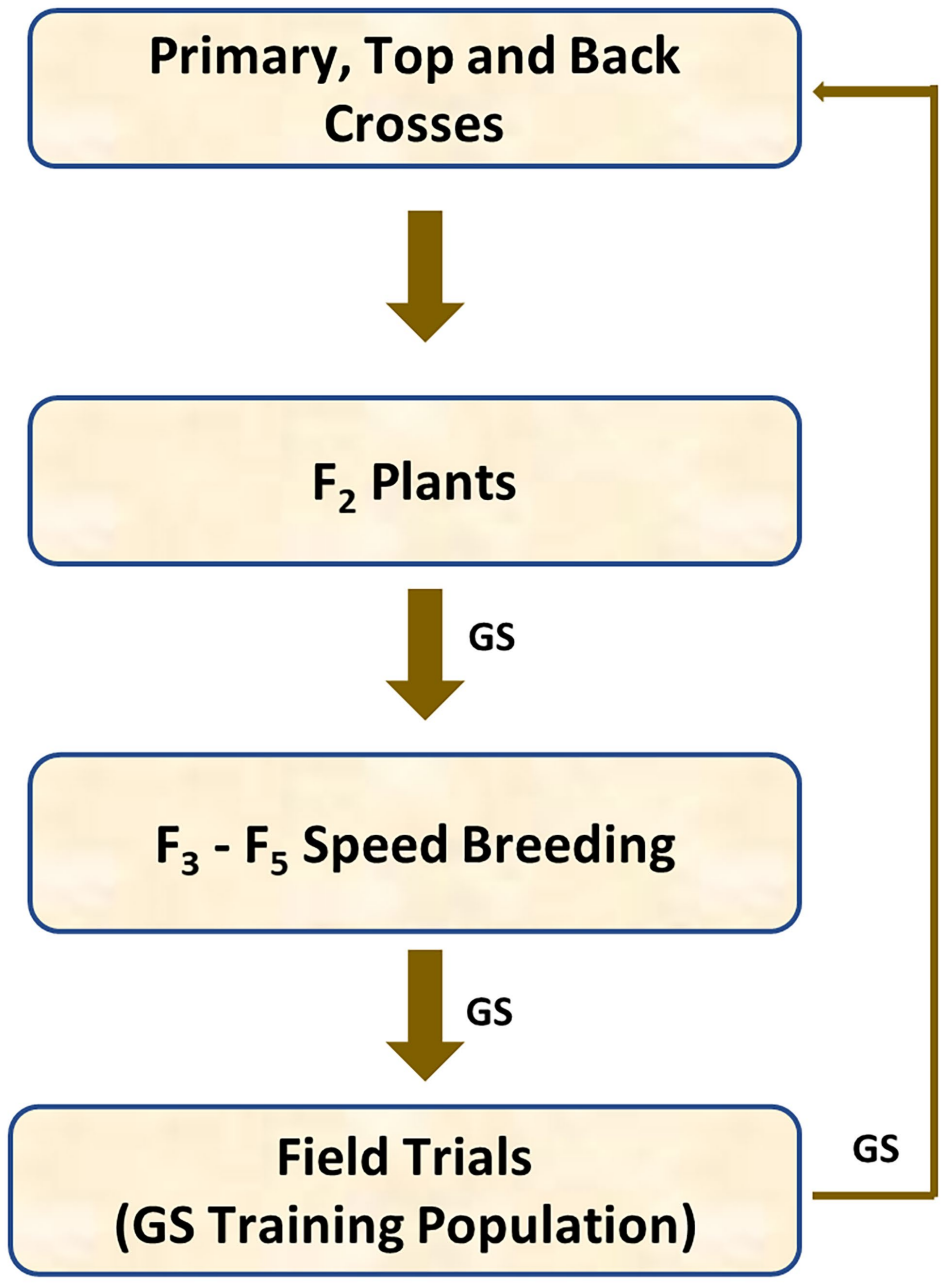
Diagram of the generic simulated cereal genomic selection (GS) breeding programme.

The generic genomic breeding programme described in Figure 1 was used for this simulation. This structure was adopted as it provides for a high level of initial introgression and high levels of recombination rather than being based on an existing breeding programme. Four hundred barley inbred lines were randomly chosen from a barley breeding population to generate 200 primary crosses, each inbred line was used once. One hundred fifty primary crosses (bi-parental crosses), whose parents had the highest total number of the favourable alleles of the major disease quantitative trait loci (QTL), were selected to generate 60 backcrosses and 90 top crosses. Therefore, 350 original crosses were formed using 400 inbred lines.

Each primary cross generated 10 F_1_ progeny, each back cross generated 10 BC_1_F_1_ progeny and each top cross generated 10 TC_1_F_1_ progeny. Each F_1_, BC_1_F_1_ and TC_1_F_1_ plant was selfed to generate 2 plants, leading to 7,000 F_2_ plants in total or 20 F_2_ plants per original family. All F_2_ plants were genotyped for genomic selection and half of them were selected on genomic estimated breeding values (GEBVs). Ten seeds were collected from each of 3,500 GEBV-selected F_2_ plants. Ten F_3_ seeds were generated from each F_2_ plant, with 35,000 F_3_ seeds in total. Single seed descent was used for breeding from F_3_ to F_5_, and 800 F_5_ plants (equally represented across families) out of 35,000 candidates were selected on GEBVs and bulked up for further testing in Stage 1 yield and disease nursery trials. Disease resistance and grain yield were evaluated in simulated field trials in two environments. Half of them were selected on GEBVs (including their own phenotypes) and used as parents of the primary crosses at the next breeding cycle.

### Simulation

Single nucleotide polymorphisms (SNP) and individuals with a call rate below 0.5 were removed from targeted genotyping-by-sequencing (tGBS) data generated for 2,139 barley breeding lines. SNPs with a minor allele frequency below 0.01 were also removed. Genomic data of 1,950 lines with 29,069 SNPs after filtering were imputed with LinkImpute (Money et al., 2015) and available for this simulation. The average number of SNPs per chromosome was 4,153 and the average length of chromosomes was 655 mb Supplementary Table 1.

**Figure 2:**
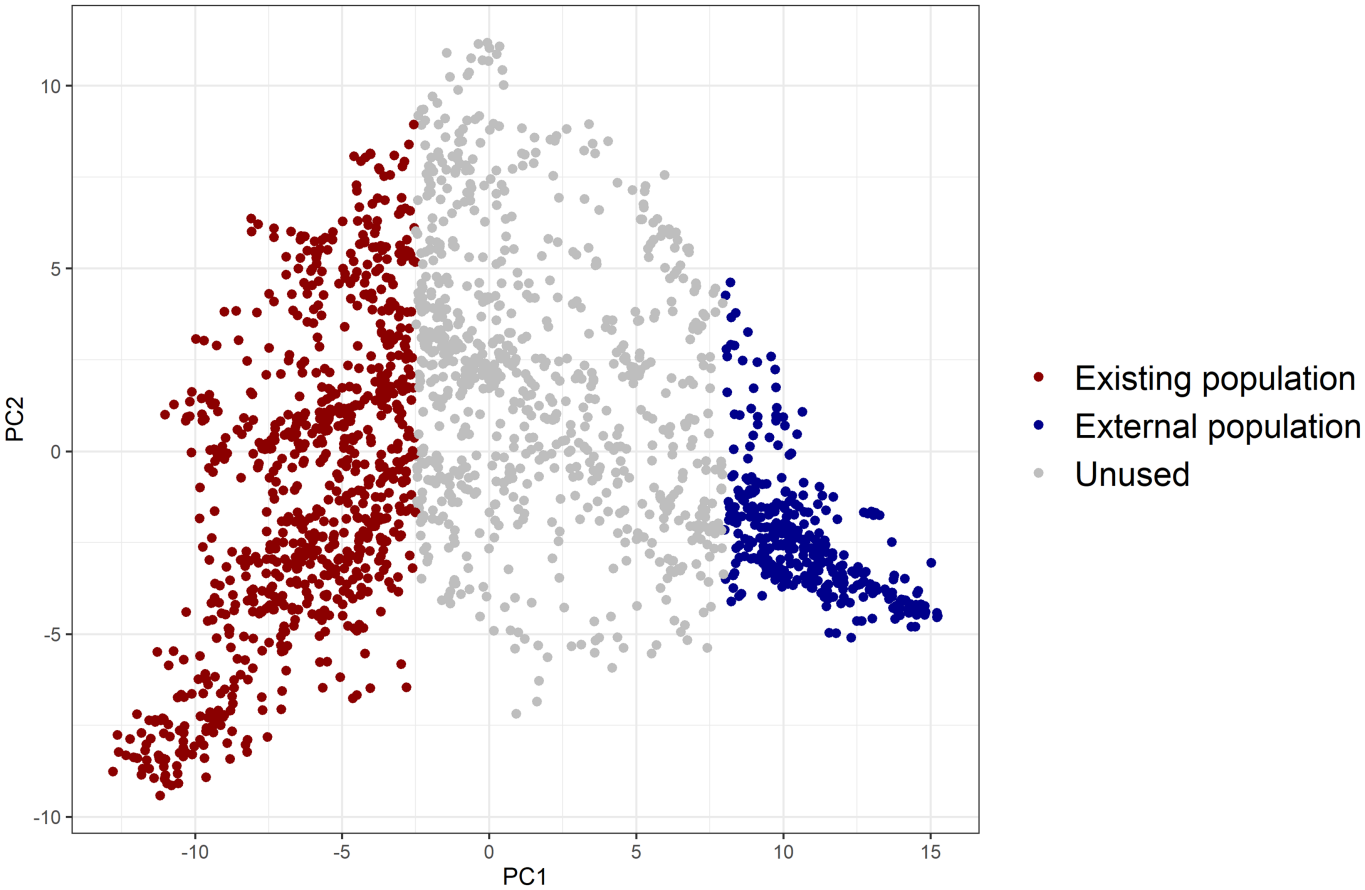
Two diverse populations created by using principal component analysis on the genomic relationship matrix of barley lines, with individuals in red colour chosen as the existing population and those in blue colour as the external population (PC1: principal component 1, PC2: principal component 2).

Two distinct and unrelated populations needed to be simulated. A principal component analysis was conducted on the genomic relationship matrix (VanRaden, 2008) of the breeding lines. We created two populations, existing and external, with large differences in allele frequencies of QTL (details given in Supplementary Table 2). The lines with the first principal component below −2.5 were used as the existing population and those with the first principal component larger than eight as the external population (Figure 2).

Two traits, disease resistance and grain yield were simulated, with narrow-sense heritabilities of 0.5 (Arabi, 2005; Xi et al., 2019) and 0.25 (Watson, 2018; Guo et al., 2020; Tsai et al., 2020), respectively, at two environments. The genetic correlation between the two environments was 0.8 for disease resistance (low level of genotype-by-environment interaction (G × E)) and 0.4 for grain yield (high level of G × E; Li et al., 2017). Each trait was controlled by approximately 1,000 QTL, each simulated as a single locus, at each environment to simulate complex genetic architecture with a number of QTL greater than the number of effective chromosome segments (Daetwyler et al., 2010). Seventy per cent of QTL controlling the same trait overlapped between two environments and there was no overlapping of QTL controlling different traits.

Quantitative trait loci for multiple traits at multiple environments were simulated in R code (details given in Supplementary Material) for a QTL overlapping rate of p between any two traits and a QTL overlapping rate of q between any two environments. Suppose k traits are simulated and the number of QTL controlling each trait in each environment is N. Within an environment, the number of QTL controlling only m traits is $Np^{m-1}{\left(1-p\right)}^{k-m}\;\mathrm{with}\;m=0,\ldots ,k$ . If k =4, numbers of QTL controlling 1, 2, 3 and 4 traits are $N{\left(1-p\right)}^{3}$ , $Np{\left(1-p\right)}^{2}$ , $Np^{2}\left(1-p\right)$ and $Np^{3}$ , respectively. Suppose w environments are simulated, the ratio of QTL having effects at s environments is


$$\frac{q^{s-1}{\left(1-q\right)}^{w-s}}{1+q}.$$

If *w* = 3, the ratios of QTL having effects at 1, 2 and 3 environments are $\frac{{\left(1+q\right)}^{2}}{1+q},\;\;\frac{q(1-q)}{1+q}$ and $\frac{q^{2}}{(1+q)}$ . For the case of this study with p =0 and q =0.7, the overlapping of QTL simulated for two traits and two environments is shown in Supplementary Table 3. There were 1,001 QTL affecting each trait in one environment and the total number QTL was 2,356.

To sample QTL effects for disease resistance (D) and grain yield (Y) at environment (A) and environment (B) with a given variance–covariance matrix


$$w=\begin{array}{cc} & D_{A}\;\;D_{B}\;\;Y_{A}\;\;Y_{B}\\ \begin{array}{c} D_{A}\\ D_{B}\\ Y_{A}\\ Y_{B} \end{array} & \left[\begin{array}{cccc} 1 & 0.8 & 0 & 0\\ 0.8 & 1 & 0 & 0\\ 0 & 0 & 1 & 0.4\\ 0 & 0 & 0.4 & 1 \end{array}\right] \end{array}$$

a matrix ( $u_{0}$ ) with four column vectors with a length of 2,356 was sampled from a normal distribution with a standard deviation of 1 and a mean of 0. Suppose $w=L^{\prime }L$ , where L is the Cholesky decomposition of w . We have $\nu =u_{0}L$ and a design matrix X linking elements in ν to QTL effects for disease resistance and grain yield at two environments (Jighly et al., 2019). The design matrix X has 2,356 rows × 4 columns, consisting of rows that were shown in the matrix x specified by the number outside the square bracket.


$$x=\left[\begin{array}{cccc} D_{A} & D_{B} & Y_{A} & Y_{B}\\ 1 & 0 & 0 & 0\\ 0 & 1 & 0 & 0\\ 1 & 1 & 0 & 0\\ 0 & 0 & 1 & 0\\ 0 & 0 & 0 & 1\\ 0 & 0 & 1 & 1 \end{array}\right]\begin{array}{c} No.ofrows\\ 177\\ 177\\ 824\\ 117\\ 117\\ 824 \end{array}$$

The additive QTL effects ( a ) for the disease at environment A and environment B , grain yield at environment A and environment B are calculated as the Hadamard (element-wise) product of X and v as a=Xv . The correlation between QTL effects was


$$\begin{array}{cc} & \begin{array}{cccc} D_{A}\;\; & D_{B} & Y_{A}\;\;\; & Y_{B} \end{array}\\ \begin{array}{c} D_{A}\\ D_{B}\\ Y_{A}\\ Y_{B} \end{array} & \left[\begin{array}{cccc} 1 & 0.79 & 0 & 0\\ 0.79 & 1 & 0 & 0\\ 0 & 0 & 1 & 0.41\\ 0 & 0 & 0.41 & 1 \end{array}\right] \end{array}$$

For disease resistance, the ten QTL with the highest effects were chosen and their effects were multiplied by a constant so that they explained 10% total genetic variance of disease resistance when 20% of inbred lines were introduced from the external population. This modelled a situation where large disease QTL may exist in population external to a breeding programme. The constant was changed based on the initial allele frequency of all disease QTL. The effects of all QTL for disease resistance were re-scaled to have a standard deviation of 1. The ten disease QTL with large effects were called major disease QTL and the rest as minor disease QTL.

The true breeding value of an individual (g) was calculated as the product of the incidence matrix and the additive genetic effects with equation g=Ma , where M is the incidence matrix related to the number of the favourable alleles of QTL. Phenotypes were the sum of the true breeding value and random residuals, which were sampled from a normal distribution with a mean of 0 and a standard deviation of $\sqrt{\frac{1-h^{2}}{h^{2}}\sigma_{a}^{2}}$ , where $h^{2}\;$ is the narrow-sense heritability of trait and $\sigma_{a}^{2}$ is the variance of true breeding value.

Within a breeding cycle, F_1_s generated in the primary crosses, top crosses and back crosses were simulated from crossing two different individuals. Individuals in the following generations (from F_2_ to Stage 1) were generated from selfing. Recombinations and mutations were simulated when gametes were generated. Recombinations were sampled from a Poisson distribution with lambda equal to 1. Mutations were sampled from a uniform distribution with a mutation rate of 0.001.

### Estimation of Marker Effects and Calculation of GEBV

Bayesian ridge regression was used to estimate marker effects, implemented using R package BGLR (de los Campos and Pérez, 2013) with equation:


y=Xβ+e

where y is a vector of phenotypes of lines, X is an incidence matrix that links with phenotypes to β the vector of marker effects, e is a vector of residuals. GEBVs (g) were predicted as the linear combination of the marker effects as in an equation:


$$g=X^{\prime }\beta$$

The accuracy of the GEBVs was estimated as the Pearson correlation between GEBVs and the true breeding values.

### Evaluation of Breeding Programme

The simulation was conducted for eight breeding cycles (each containing different generations: each containing: the primary crosses, F_2_, …, F_5_ and field trials) with 50 replicates for each scenario. Phenotypic selection was conducted at breeding cycles 1–3 and genomic selection conducted at breeding cycles 4–8. The training population consisted of individuals chosen from the latest three breeding cycles, with 400 phenotyped individuals at each breeding cycle. Lines were selected with a simple selection index (I) :


$$I=w_{D}\times \frac{u_{D}}{\sigma_{u_{D}}}+w_{Y}\times \frac{u_{Y}}{\sigma_{u_{Y}}}$$

where $u_{D}$ and $u_{Y}$ are the vectors of GEBVs in genomic selection and phenotypic values in phenotypic selection, $\sigma_{u_{D}}$ and $\sigma_{u_{Y}}$ the standard deviations of GEBVs or phenotypic values, $\;w_{D}$ and $w_{Y}$ index weights on disease resistance and grain yield, respectively, and $w_{D}+w_{Y}=1$ . Trait index weights varied between scenarios.

The genetic gain achieved in a breeding programme and the predictive accuracy of GEBVs were evaluated. The genetic gain was defined as the average true breeding values of individuals selected as parents for the next breeding cycle, expressed in the unit of the additive genetic standard deviation $\left(\sigma_{a}\right)$ of the start of the simulation at the beginning of breeding cycle 1. The prediction accuracy of GEBVs was defined as the Pearson correlation between GEBVs and true breeding values. When evaluating the prediction accuracy of GEBVs, phenotypes of individuals to be ranked were not used in estimating marker effects, which means that GEBVs were calculated using marker effects estimated at the previous breeding cycle.

### Simulation Scenarios

The first group of scenarios were to investigate the effect of the level of minor disease QTL frequency in the existing programme on the benefit of introgression. Three levels of minor disease QTL frequencies were tested: high, medium and low, with an average frequency of the favourable alleles of minor disease QTL of 0.85, 0.52 and 0.27, respectively (Supplementary Table 2). Introgression of external resources was conducted only once at the beginning of genomic selection at breeding cycle 4. There were no major disease QTL in the existing population before their introduction. The average frequency of the major disease QTL in the external resources was 0.65. After using external lines as primary crossing parents at 20% into the existing population and further increasing the major disease allele frequency through top and back crosses, the average frequency of the major disease QTL reached 0.13 for all three levels of minor disease QTL frequency groups at the beginning of breeding cycle 4. The average frequency of the minor disease QTL and that of yield QTL declined slightly after the introduction of the external lines. To meet the criterion of 10% variation explained by the major disease QTL when introducing 20% external lines, the size of the mean major disease QTL effect was set at 3.21, 4.70 and 3.34 for the three levels of the minor disease QTL frequencies, respectively. Grain yield QTL were sampled from a normal distribution so that their mean magnitude was zero. Disease QTL were also sampled from a normal distribution and ten QTL with the largest effects were rescaled as the major disease QTL so that the mean magnitude of the minor disease QTL was a little bit below zero and the mean magnitude of the major disease QTL had positive values.

The second group of scenarios were used to investigate the strategies that potentially could accelerate the introgression of external lines. In this group, the base scenario had a high frequency of the minor disease QTL, the size of the major disease QTL was equal to 3.21, equal index weights were applied on disease resistance and grain yield ( $w_{D}=w_{Y}=0.5$ ), the index did not include the number of the favourable alleles of the major disease QTL, and 20% external lines were introduced. Three scenarios were conducted to compare the benefit from introducing external lines by modifying one of these parameters at a time:

- Scenario 1, various percentages of external lines introgressed.

Apart from 20% external lines introgressed, scenarios with 1, 5, 10% external lines introgressed were examined. External lines were chosen to maximise the number of favourable alleles of the major disease QTL. The external lines with the highest number of the favourable alleles of the major disease QTL were chosen as a part of parental lines for primary crosses, top crosses and back crosses. The initial frequencies of the favourable alleles of the major disease QTL were 0.78, 0.75, 0.72 and 0.65 among the external lines introduced and 0.01, 0.04, 0.07 and 0.13 among parents after introduction.

- Scenario 2, increased major disease QTL effect size.

The mean effect size of major disease QTL was increased from 3.21 to 5.20.

- Scenario 3, different selection pressure on disease resistance.

Two options of changing selection pressure on disease resistance were investigated: changing index weights and including the number of the favourable alleles of the major disease QTL in the selection index. In the first option, a higher index weight was applied to disease resistance: 70% selection pressure on disease resistance and 30% selection pressure on grain yield ( $w_{D}=0.7\;\mathrm{and}\;w_{Y}=0.3$ ). In the second option, equal weights were applied on both traits but the number of favourable alleles of the major disease QTL was added into the selection index using the equation below:


$$I=w_{D}\times \frac{u_{D}}{\sigma_{u_{D}}}+w_{Y}\times \frac{u_{Y}}{\sigma_{u_{Y}}}+kN$$

where N the total number of favourable alleles of the major disease QTL, k a constant of value of 0.01, and $w_{D}=w_{Y}=0.5$ . The sum of the first two terms of index I has an expected variance of 0.5 with a standard deviation of 0.7. N has a value ranging from 0 to 20 inclusively and kN has a value ranging from 0 to 0.2. The value of k was decided by comparing different values between 0.005 and 0.1.

## Results

### Level of Minor Disease QTL Frequency

**Figure 3:**
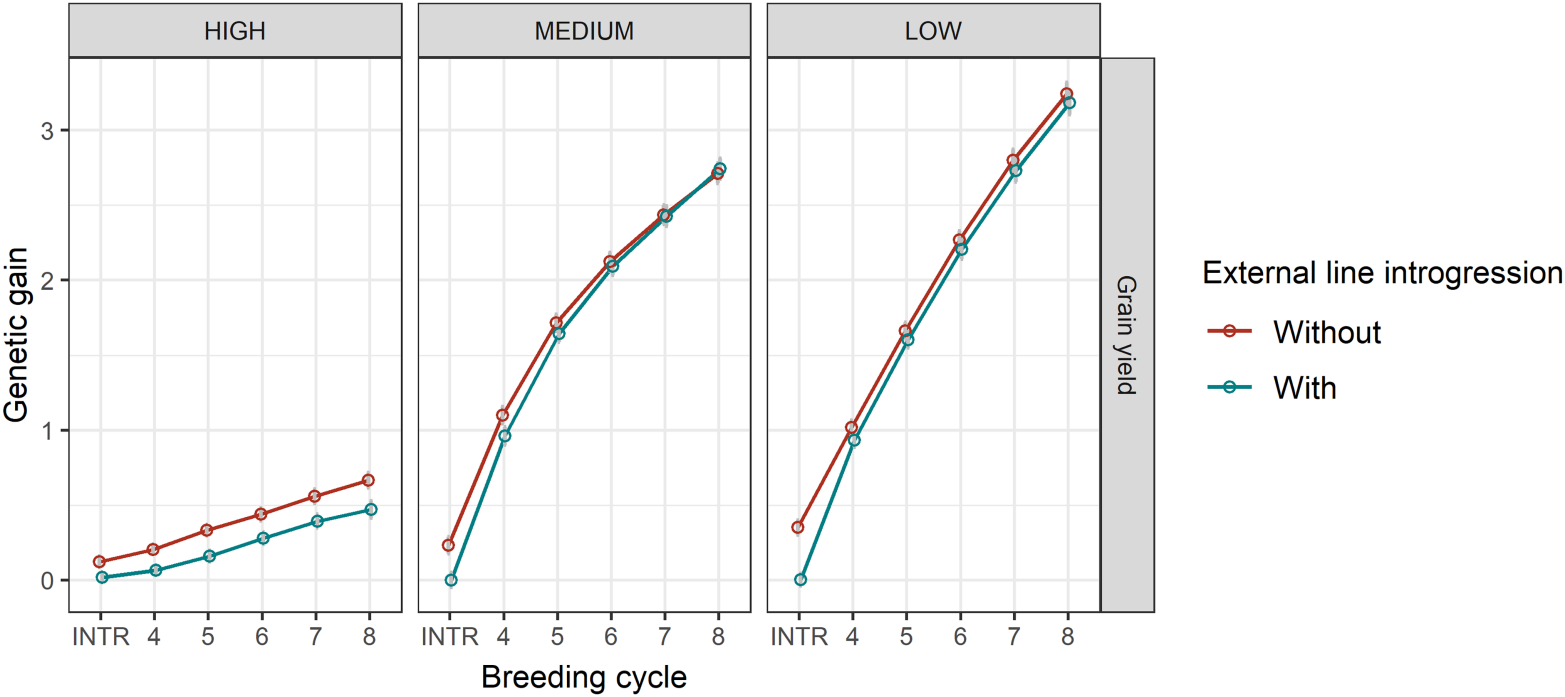
Genetic gain for disease resistance and grain yield in genomic selection without and with 20% external resources introduction at the beginning of breeding cycle 4 in the cases of high (HIGH), medium (MEDIUM) and low (LOW) average frequencies of the minor disease QTL. Equal index weights for disease resistance and grain yield were used in the selection index. Genomic selection was conducted at breeding cycles 4–8 and introgression occurred once at the beginning of breeding cycle 4. INTR indicates the performance of parents at breeding cycle 4 after introgression. The standard error of genetic gain among replicates of 50 simulations is shown with error bars but in some cases are very small.

**Figure 4:**
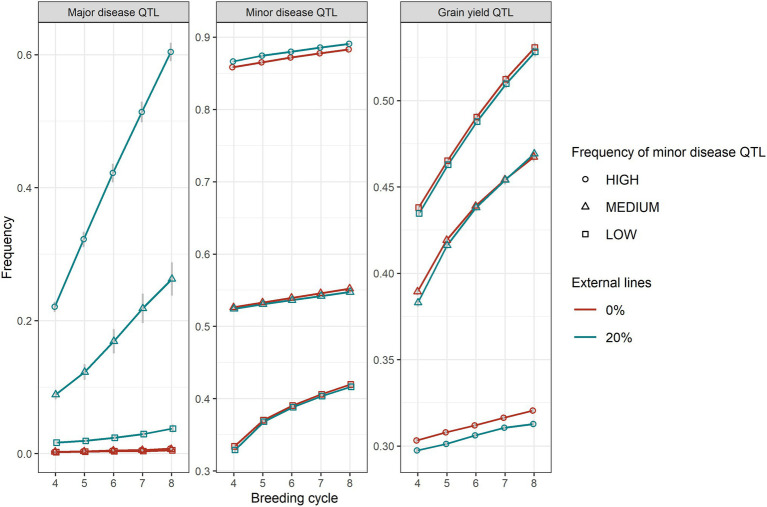
The average frequencies of the favourable alleles of major disease QTL, minor disease QTL and grain yield QTL between GS with (20%) and without (0%) of external resources introduced for high, medium, and low frequencies of the minor disease QTL. Equal index weights were used in the selection index. The standard errors of QTL allele frequencies among replicates of 50 simulations are shown with error bars but in some cases are very small.

The level of the average initial allele frequency of the minor disease QTL influenced how beneficial the introgression of external resources was for a breeding programme (Figure 3). When the initial average frequency of the minor disease QTL was high, introgression significantly increased genetic gain for disease resistance, due to a large increase in the favourable allele frequency of the major disease QTL and a slightly increase in the favourable allele frequency of the minor disease QTL (Figure 4). The superiority of introgression for disease resistance over selection without introgression increased in later breeding cycles. In the case of a medium initial frequency of the minor disease QTL in the existing programme, the superiority of introgression was reduced but the trend for disease resistance remained similar. This increase of genetic gain in disease resistance stemed from the increase of the favourable allele frequency of the major disease QTL. When the initial average frequency of the minor disease QTL was low, introgression led to less genetic gain for disease resistance than genomic selection without introgression at the beginning of the selection and a similar level of genetic gain in later cycles. In this case, introgression slightly increased the favourable allele frequency of the major disease QTL at all breeding cycles, but slightly reduced the favourable allele frequency of the minor disease QTL at the first breeding cycle on introgression. When the minor disease QTL had a high initial average frequency, introgression led to a lower genetic gain for grain yield (linkage drag) across cycles due to lower allele frequency of the grain yield QTL. When minor disease QTL frequency was medium or low, linkage drag was observed at early breeding cycles and diminished completely over time for the medium scenario and was small but constant for the low scenario (Figure 3).

### Percentage of Introgressed External Resources

**Figure 5:**
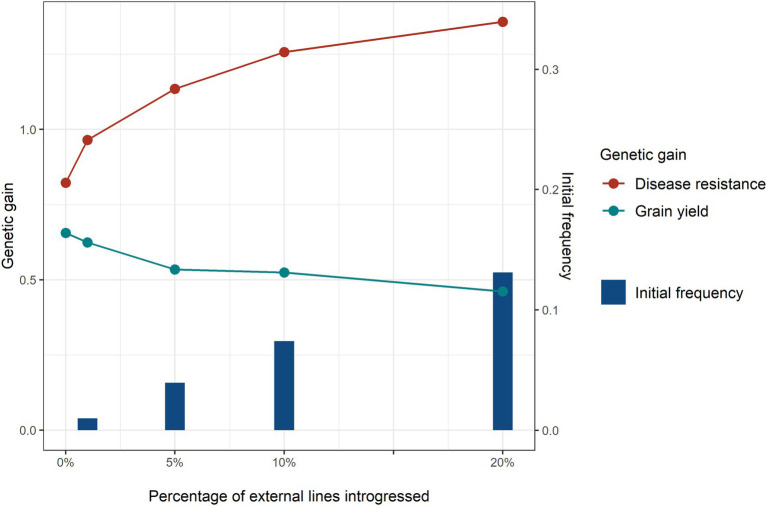
Genetic gain of disease resistance (red line) and grain yield (turquoise line) obtained with different levels: 0, 1, 5, 10, and 20% of external resource introgression at breeding cycle 4 and initial frequency (blue bar) of the favourable alleles of the major disease QTL at the beginning of selection. Equal index weights on disease resistance and grain yield were used in the selection index. Genomic selection was conducted at breeding cycles 4–8 and introgression occurred once at the beginning of cycle 4.

The results in this section and the following sections are all compared using a base scenario with a high minor disease QTL frequency. Introgressing a higher percentage of external sources increased genetic gain for disease resistance (Figure 5). It seemed that any percentage of major disease QTL introgressed to the breeding programme led to a benefit for disease resistance. Even for 1% external lines introgressed, which is the equivalent to only 4 external lines and 0.01 initial favourable allele frequency of the major disease QTL, considerable genetic gain in disease resistance was achieved. But the incremental advantage from introgression diminished at a higher percentage of introgression. The benefit in genetic gain of disease resistance was not proportional to the initial favourable allele frequency of the major disease QTL at the beginning of the introgression. Genetic drag was observed at all levels of introgression, where higher levels of introduction lead to greater reductions in genetic gain for grain yield. At low levels of external parental usage, the magnitude of linkage drag on grain yield was much smaller than the benefit for disease resistance. For instance, with a benefit of introgression for disease resistance of 0.14 genetic standard deviations and a linkage drag for grain yield of 0.03 genetic standard deviation when introgressing 1% of external lines.

### Effect Size of Major Disease QTL

**Figure 6:**
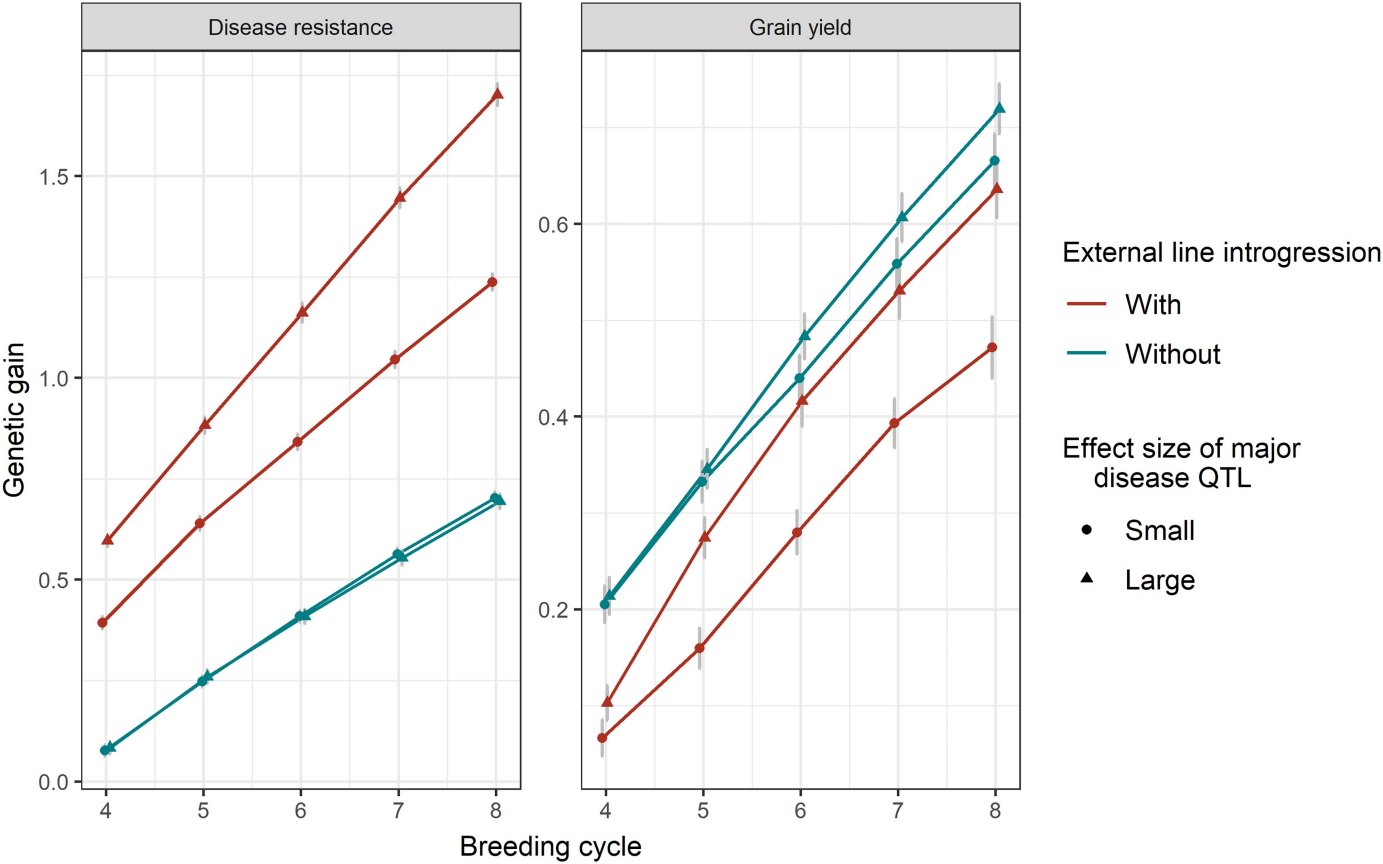
Genetic gain of disease resistance and grain yield obtained from a genomic selection with 0% or 20% introduction of external resources with small (circle shape) and large (triangular shape) sizes of the major disease QTL. Equal index weights on disease resistance and grain yield were used in the selection index. Genomic selection was conducted at breeding cycles 4–8 and introgression occurred once at the beginning of breeding cycle 4. The standard errors of genetic gain among replicates of 50 simulations are shown with error bars but in some cases are very small.

Larger effect sizes of the major disease QTL introduced led to more genetic gain in disease resistance (Figure 6). As expected, the genetic gain obtained from scenarios without introgression was the same between small and big sizes of the major disease QTL, because they were not present in the existing population. The genetic drag in grain yield was reduced when the major disease QTL effect sizes were larger. Without introgression, there was no significant difference in the allele frequencies of major disease QTL, minor disease QTL and yield QTL between two different sizes of the major disease QTL. With introgression, a larger size of the major disease QTL led to a higher frequency of major disease QTL and yield QTL and a lower frequency of the minor disease QTL (Supplementary Figure 1).

### Changing Selection Pressure on Disease Resistance

**Figure 7:**
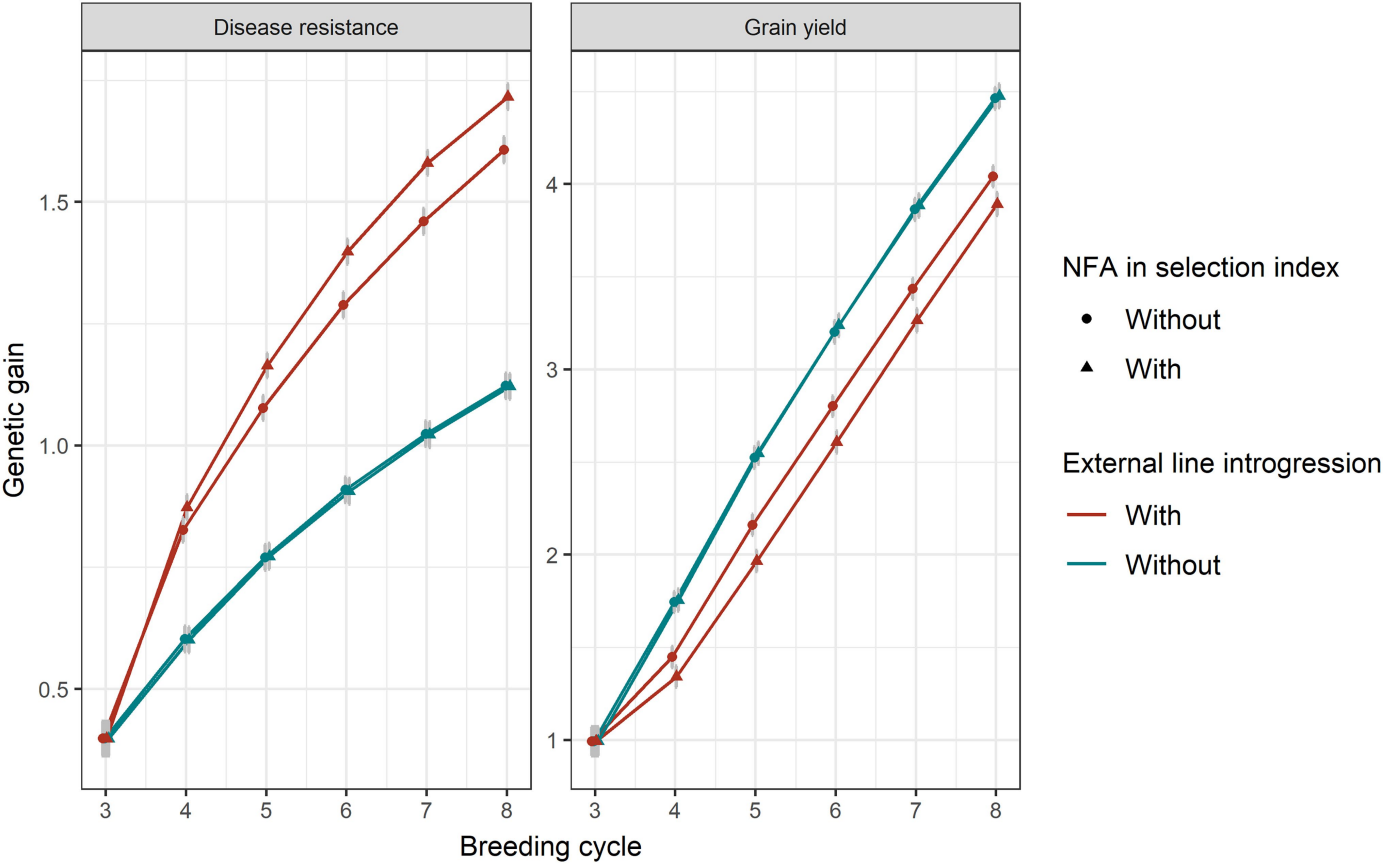
Genetic gain for disease resistance and grain yield without or with 20% introduction of external resources with (triangular shape) or without (circle shape) including the number of favourable disease alleles in selection index. Equal index weights (0.5) for both traits were used in the selection index plus 0.01 of weight on the number of favourable disease alleles (NFA). Genomic selection was conducted at breeding cycles 4–8 and introgression occurred at the beginning of breeding cycle 4. The standard errors of genetic gain among replicates of 50 simulations are shown with error bars but in some cases are very small.

Genetic gain in disease resistance increased when a higher index weight was applied on disease resistance, which, in turn, resulted in greater linkage drag for grain yield (Supplementary Figure 2). The inclusion of the number of favourable major disease alleles in the index to provide additional selection pressure on the loci themselves rather than on the trait only was favourable for disease resistance (Figure 7). However, this also caused more linkage drag in grain yield.

## Discussion

### Level of Allele Frequency of Minor Disease QTL

Introgression introduces novel variation into a breeding programme. This study dynamically simulated a cereal breeding programme with introgression of external resources using stochastic simulation, which allows us to examine the process of cereal genomic selection in detail. The initial aim was to understand the potential benefits from introgression of external but somewhat adapted lines to a breeding programme if external QTL or major genes explained some of the genetic variation. The first finding of this study was that the benefit of introducing external lines depended strongly on the level of existing variation for the trait of interest in the breeding programme. Introgression was only beneficial when the existing variation was almost fully exploited in the breeding programme. There is a competitive relationship between the major genes to be introgressed and minor genes in the existing breeding programme. When the existing QTL have low allele frequencies, selection increases their allele frequencies and the variation explained by these QTL increased substantially due to the large number minor QTL. Therefore, no extra benefit could be obtained from introgression of external resources. On the other hand, when the existing QTL have high allele frequencies, the variation explained by these QTL decreases with increasing frequencies and introgression of external major genes is beneficial. In real life of plant breeding, it may be hard to know the initial allele frequency of QTL controlling a quantitative trait. A practical way to check if an existing breeding programme needs introgression of external resource is to investigate genetic variation of the trait of interest in the breeding programme. If the variation is very low, introgession of external resources will benefit the trait. It is safe to deduce that introgression of external lines will be beneficial in an extreme situation where there is no variation in a disease of interest, i.e., no resistance at all, in the existing breeding programme. For example, when a new pathotype emerges, there may be no resistance to the pathotype, introgression of genes/QTL from an elite germplasm that possesses resistance genes/QTL to the pathotype will be beneficial to the breeders.

### Genomic Selection as a Tool of Introgression

The implementation of high-density maps and reference genome technology enable more focused introgressions and paves the way to better exploitation of novel exotic alleles, genes and QTL (Klymiuk et al., 2019). The development of genomic tools to facilitate the selection of desirable genotypes, introgression breeding is returning as a mainstream activity (Prohens et al., 2017; Hao et al., 2020). Genomic selection is a useful strategy for rapid introgression of exotic germplasm in an existing breeding programme (Bernardo, 2009; Odegård et al., 2009a,b). Genomic selection also speeds up the process of introgression of a gene, simultaneously increasing genetic gain (Gaspa et al., 2015). The current study confirmed that genomic selection is a powerful tool for introgressing genes/QTL from outside a breeding programme. With genomic data available, the favourable alleles of external novel genes can be accurately predicted. Even when the average allele frequency of the major disease QTL was only 0.01 for 1% external lines introduced, introgression led to a remarkable benefit for disease resistance (Figure 5). Besides, the benefit of introgressing external resources did not show a linear relationship with the number of lines introgressed and the marginal benefit reached its maximum at 5% introgression. This finding suggests that there might be an optimal point of number of lines introgressed in a breeding programme. However, this optimal level of introgression is difficult to determine in real breeding programmes, because it is affected by many factors, such as population structure and size, number of crosses and offspring, trait heritabilities, distribution of allele frequencies, and breeding schemes. In addition, the genomic prediction method could also potentially affect the efficiency of introgression, especially when the focus is on few large QTL. This study applied Bayesian ridge regression which weights all loci equally (de los Campos et al., 2013). Methods that can increase weights on loci with larger effects (e.g., BayesR, BayesB), in this case the 10 major disease QTL, could potentially increase introgression efficiency further and may reduce linkage drag in other traits.

### Linkage Drag

The introgression of favourable alleles for one trait often introduces inferior alleles for other traits due to the linkage disequilibria between favourable and inferior alleles and also the linkage drag of, unlinked, inferior alleles in the genetic background. Linkage drag can be reduced by reducing the size of the chromosome segments carrying the novel genes, which reduces the introduction of linked inferior genes (Hospital, 2005; Hernandez et al., 2020), and reducing the proportion of the external germplasm in the genetic background. Marker-aided backcrossing has been used to reduce linkage drag in disease resistance, malting quality and grain yield (Jefferies, 2000; Matus et al., 2003; von Korff et al., 2008; Peng et al., 2014). Linkage drag was also reduced by an increasing number of back cross generations, marker data points needed and total population sizes across generations (Peng et al., 2014). Nevertheless, backcrossing strategies become complex when the several new loci need to be introgressed and reduce the possibility of new, novel and favourable “background” alleles being incorporated into the breeding germplasm pool. A few ways of accelerating introgression were proposed in this study. A balance needs to be considered between quick integration of the external novel trait and linkage drag in other traits. Linkage drag of traits in the existing breeding programme caused by introgression of novel genes from external resources is a concern in plant breeding (Prohens et al., 2017; Hao et al., 2020). Novel genes or QTL are normally introgressed from exotic populations, such as wild relatives, landraces or individuals from other breeding programmes. The more genetically different the external lines are to the existing breeding programme, the higher the likelihood of finding novel genes, but it is also more likely to have linkage drag in other traits. In the current study, genomic selection provided two ways of affecting the linkage drag in grain yield. One way was to only introgress major disease QTL with larger effect sizes. The linkage drag in grain yield was reduced in selection with a larger size of the major disease QTL. The reason might be that when major disease QTL had larger effects, selection pressure for disease resistance was mainly on the major disease QTL and selection pressure on grain yield increased. The evidence of this phenomenon was that a big reduction in the frequencies of the minor disease QTL and a big increase in the frequencies of grain yield QTL were found when the size of major disease QTL increased, in the case of introducing 20% external lines. If linkage drag is a major concern, introgression may want to focus on few large external QTL as the introduction of a large number of QTL with antagonistic effects on other traits will lead to linkage drag due to the limits on recombination in each generation to break up unfavourable combinations. The impact on grain yield could be addressed by reducing the speed of introgressing major QTL in the population by reducing the index weights on the major QTL.

### Limitations

The breeding lines used in this simulation were taken from a barley breeding population in Australia. It needs to be pointed out that those breeding lines are under intensive selection and well-adapted in the environments in Australia. Lines in the existing and the external populations were identified based on the results of the principal component analysis. The two populations are quite different in allele frequencies to some extent, 0.65 for the major disease QTL, 0.03–0.19 for the minor disease QTL and 0.05–0.13 for grain yield QTL, but may still be more similar compared to landrace cultivars. Nevertheless, the conclusions are still valid if external resources are taken from more genetically different germplasm. The performance of the novel trait introgressed would be high but that of other traits may be low. In this case, extra benefit of the novel trait is even higher but more linkage drag may occur. Genomic selection facilitated pre-breeding that incorporates favourable loci from landraces into more elite genetic backgrounds would also be expected to reduce linkage drag (Gorjanc et al., 2016). It might be worth to conduct further research on introgression of breeding material from landrace or pre-breeding material, in order to fill a knowledge gap in this area.

Some studies observed a negative correlation between grain yield and disease resistance (Brown, 2002). For example, doubled-haploid barley progeny of crosses between three *mlo* mutant lines and susceptible cultivars were grown in trials in which foliar diseases, including mildew, were controlled with fungicides. Mildew-resistant (*mlo*) lines had an average grain yield that was 4.2% lower than that of mildew-susceptible (*Mlo*) lines (Brown, 2002). In this study, disease resistance and grain yield were assumed to be uncorrelated. If disease resistance has a negative genetic correlation with grain yield, a higher linkage drag may be expected in grain yield with introgression of major disease QTL. In the future, it would be worthwhile to investigate introgression when pleiotrophy exists.

The introgression of ten QTL explaining 10% of disease variation was simulated in this study. It is common that several larger effect disease QTL exists, for example wheat rust disease (Mago et al., 2005; Nsabiyera et al., 2016; Zhang et al., 2017). It is also known that these traits generally have many minor QTL affecting the trait and these are an important part of trait variation (Joukhadar et al., 2020). If major QTL explain more or all of the variation in a trait, introgression would behave similar to our case of high frequency in minor disease QTL. In an even more extreme case with very few QTL conferring complete resistance, introgression would of course be highly sucessful and linkage drag could likely be completely avoided with a targeted backcross strategy.

The current simulation was conducted with a one-time introgression. Future work may need to test if multiple introgressions would change trends. In principle, the results obtained are only expected to be more extreme with additional introgression events. That is higher genetic gain in disease, subject to diminshing returns at greater introgression levels, and more linkage drag in grain yield. In addition, the minor disease QTL were simulated to be present in both existing and external populations. Further investigation might also need to assume that only some of the large disease QTL were shared between two populations.

There were three different types of crosses in this study: bi-parental crosses, backcrosses and top crosses. However, the effects of these crossing strategies were not investigated in this study. Introgression *via* a bi-parental cross versus backcrosses to the recurrent parent have different effects on linkage drag or genetic gain for the trait of interest and also depend on the relative genetic levels of donor and elite germplasm (Allier et al., 2019). It will be an interesting topic for the future research to investigate the effects of crossing strategies on the genetic gain of disease resistance and linkage drag in other traits when introgressing external breeding material in an advanced breeding programme.

Pleiotrophy is a common property of major genes and is a source of correlation between traits (Falconer and Mackay, 1996). It has been reported that grain yield is correlated with the resistance to diseases, such as yellow rust, leave rust, powdery mildew and fusarium head blight, in the literature (Singleton et al., 1982; Zetzsche et al., 2020). Therefore, there should be some QTL that have pleiotropic effects and affect both traits. For the simplicity of simulation, QTL simulated did not have pleiotropic effects, i.e., one QTL only controlled one trait. In the future study, it is worth simulating pleiotropic effects to investigate strategies of introgressing external resources under the circumference where some QTL control multiple traits.

## Conclusion

Introgression of external resources into a breeding programme was only beneficial when the breeding programme had already exploited its variation in the desired trait or lacked variation completely for the trait. Genomic selection is effective at introgressing multiple large QTL. Several strategies are able to increase this efficiency, however, only concentrating on the introgression of large effect QTL was able to partially avoid linkage drag in another traits. Plant breeders are advised to carefully evaluate the variation present in the germplasm for target traits before embarking on introgression of external lines.

## Data Availability Statement

The datasets presented in this study can be found online here: https://datadryad.org/stash with DOI: https://doi.org/10.5061/dryad.70rxwdc15.

## Author Contributions

YL ran the simulation, performed the analyses, and drafted the manuscript. FS and ZL developed the basic simulation modules. HR, DMo, AR, JG, and DMu provided information of cereal breeding programme parameters. GK-G, MH, and JT genotyped and imputed the tGBS genomic dataset. YL, HR, AR, MH, JT, and HD designed the study and assisted with drafting the manuscript. All authors read and approved the final copy of the manuscript.

## Funding

This study was funded by InterGrain Pty Ltd and the Agriculture Victoria Services Pty Ltd.

## Conflict of Interest

YL, FS, ZL, GK-G, MH, JT, and HD were employed by the company Agriculture Victoria Services Pty Ltd. HR, DMo, AR, JG, and DMu were employed by the company InterGrain Pty Ltd.

## Publisher’s Note

All claims expressed in this article are solely those of the authors and do not necessarily represent those of their affiliated organizations, or those of the publisher, the editors and the reviewers. Any product that may be evaluated in this article, or claim that may be made by its manufacturer, is not guaranteed or endorsed by the publisher.

## Footnotes

^1^ https://www.barleyaustralia.com.au/industry/barley/

## Supplementary Material

Supplementary Figure 1 Favourable allele frequencies of major disease QTL, minor disease QTL and yield QTL for with or without 20% introduction of external resources with small (circle shape) and large (triangular shape) effect sizes of the major disease QTL. Equal index weights on disease resistance and grain yield were used in the selection index for disease resistance and grain yield. Genomic selection was conducted at breeding cycles 4–8 and introgression was conducted once at the beginning of breeding cycle 4. The standard errors of genetic gain among replicates of 50 simulations are shown with error bars but some errors are small.

Supplementary Figure 2 Genetic gain for disease resistance and grain yield without or with 20% introgression of external resources with equal index weight on disease resistance ( $w_{D}$ =0.5) and high index weight on disease resistance ( $w_{D}$ =0.7). Index weight for grain yield was 0.5 when $w_{D}$ =0.5 and 0.3 when $w_{D}$ =0.7. Genomic selection was conducted at breeding cycles 4–8 and introgression was conducted once at the beginning of breeding cycle 4. The standard errors of genetic gain among replicates of 50 simulations are shown with error bars but some errors are small.
