# Supplementary material for: Benefit of Introgression Depends on Level of Genetic Trait Variation in Cereal Breeding Programmes
Source: Front Plant Sci. 2022 Jun 15;13:786452. doi: 10.3389/fpls.2022.786452 (PMC9240786; doi:10.3389/fpls.2022.786452)
Supplement: Supplementary Figure 1 — Favourable allele frequencies of major disease QTL, minor disease QTL and yield QTL for with or without 20% introduction of external resources with small (circle shape) and large (triangular shape) effect sizes of the major disease QTL. Equal index weights on disease resistance and grain yield were used in the selection index for disease resistance and grain yield. Genomic selection was conducted at breeding cycles 4–8 and introgression was conducted once at the beginning of breeding cycle 4. The standard errors of genetic gain among replicates of 50 simulations are shown with error bars but some errors are small. [file Data_Sheet_1.docx]

**Supplementary Table 1**. Distribution of SNPs across chromosomes

| Chromosome | No. of SNPs | Length (mbp) |
| --- | --- | --- |
| 1 | 2811 | 558.53 |
| 2 | 5446 | 767.61 |
| 3 | 4437 | 699.59 |
| 4 | 3469 | 646.52 |
| 5 | 5031 | 669.42 |
| 6 | 3659 | 583.10 |
| 7 | 4216 | 656.74 |

**Supplementary Table 2**. Mean frequencies of QTL for existing and external populations and the mean QTL effects for major disease, minor disease, and yield QTL for three schemes of the one-time introduction of external lines

| Frequency of minor disease QTL | QTL category | Favourable allele frequency | | Introducing 20% | Mean magnitude of QTL effect |
| --- | --- | --- | --- | --- | --- |
|  |  | Existing | External |  |  |
| High | Major disease | 0 | 0.65 | 0.13 | 3.21 |
|  | Minor disease | 0.85 | 0.88 | 0.86 | -0.02 |
|  | Grain yield | 0.30 | 0.25 | 0.30 | 0 |
| Medium | Major disease | 0 | 0.65 | 0.13 | 4.70 |
|  | Minor disease | 0.52 | 0.50 | 0.52 | -0.03 |
|  | Grain yield | 0.30 | 0.24 | 0.29 | 0 |
| Low | Major disease | 0 | 0.65 | 0.13 | 3.34 |
|  | Minor disease | 0.27 | 0.08 | 0.24 | -0.03 |
|  | Grain yield | 0.41 | 0.28 | 0.40 | 0 |

**Supplementary Table 3.** Number of QTL for the two traits at two environments and their overlap between environments. There were no overlapping QTL between traits.

| Trait | Environment | No. of QTL | QTL Overlapping |
| --- | --- | --- | --- |
| Disease resistance | 1 | 177 | Only having effects at environment 1 |
| Disease resistance | 2 | 177 | Only having effects at environment 2 |
| Disease resistance | 1 & 2 | 824 | Having effects at both environments |
| Grain Yield | 1 | 177 | Only having effects at environment 1 |
| Grain Yield | 2 | 177 | Only having effects at environment 2 |
| Grain Yield | 1 & 2 | 824 | Having effects at both environments |
