# Supplementary material for: Benefit of Introgression Depends on Level of Genetic Trait Variation in Cereal Breeding Programmes
Source: Front Plant Sci. 2022 Jun 15;13:786452. doi: 10.3389/fpls.2022.786452 (PMC9240786; doi:10.3389/fpls.2022.786452)
Supplement: Supplementary Figure 2 — Genetic gain for disease resistance and grain yield without or with 20% introgression of external resources with equal index weight on disease resistance ( wD =0.5) and high index weight on disease resistance ( wD =0.7). Index weight for grain yield was 0.5 when wD =0.5 and 0.3 when wD =0.7. Genomic selection was conducted at breeding cycles 4–8 and introgression was conducted once at the beginning of breeding cycle 4. The standard errors of genetic gain among replicates of 50 simulations are shown with error bars but some errors are small. [file Data_Sheet_2.docx]

# Supplementary R Script

## R scripts for simulation of QTL for multiple traits across multiple environments

#***************************************************************

# This script is used to simulate QTL for multiple traits

# across multiple environments when providing the correlations

# between traits and environments and the percentages of overlapping

# between traits and between environments. It can simulate QTL

# for four traits and three environments in maximum.

#

# Author: Yongjun Li (Yongjun.Li@agriculture.vic.gov.au)

#***************************************************************

rm(list=ls())

#***************************************************************

# Generating QTL groups per environments

#***************************************************************

environ_combination <- function(nsites){

x = 1:nsites

comb <- data.frame(matrix(0,2**nsites-1,nsites))

k <- 1

for (i in 1:nsites) {

a <- t(combn(x=nsites,i))

for (j in 1:dim(a)[1]){

for (m in 1:dim(a)[2]){

comb[k,a[j,m]] <- 1

}

k <- k + 1

}

}

names(comb) <- paste0('site',1:nsites)

return(comb)

}

#***************************************************************

# Generating QTL groups for multipe environments

#***************************************************************

qtl_per_environment <- function(nt,nqtl,traitoverlap){

grp <- as.data.frame(matrix(0,2^nt,nt+2))

names(grp) <- c(paste('t',seq(1:nt),sep=''),'N','ctrl')

nrows <- dim(grp)[1]

vec <- data.frame(a=2^(seq(1:nt)-1))

vec

for (i in 1:nt){

grp[,i] <- rep(c(rep(0,vec$a[i]),rep(1,vec$a[i])),vec$a[nt+1-i])

}

for (i in 1:nrows){

for (j in 1:nt){

if (grp[i,j]!=0){

grp$ctrl[i] <- j

break

}

}

}

for (i in 1:nrows){

grp$N[i] <- nqtl

for (j in 1:nt){

if (j==grp$ctrl[i]) {

grp$N[i] <- grp$N[i]

} else {

if (grp[i,j]==0){

grp$N[i] <- round(grp$N[i]*(1-traitoverlap))

} else {

grp$N[i] <- round(grp$N[i]*traitoverlap)

}

}

}

}

grp$nt <- 0

for(i in 1:dim(grp)[1]) grp$nt[i] <- sum(grp[i,1:nt])

grp$string <- ''

for (i in 1:nt){

grp$string <- paste(grp$string,grp[,i],sep='')

}

return (grp)

}

#***************************************************************

# Generating QTL groups combining traits and environments

#***************************************************************

group_combining <- function(ntraits,nsites,nqtl,traitoverlap,envoverlap)

{

qtlGrp <- qtl_per_environment(nt=ntraits,nqtl=nqtl,

traitoverlap=traitoverlap)

qtlGrp <- qtlGrp[qtlGrp$nt>0,]

envcomb <- environ_combination(nsites)

envcomb$sum <- rowSums(envcomb)

combgrp <- as.data.frame(matrix(0,dim(qtlGrp)[1]*dim(envcomb)[1],

ntraits*nsites+1))

names(combgrp) <- c(paste('t',1:(ntraits*nsites),sep=''),'N')

head(combgrp)

overlapped <- 2*envoverlap/(1+envoverlap)

nonoverlapped <- (1-envoverlap)/(1+envoverlap)

k <- 1

for (i in 1:dim(qtlGrp)[1]){

set <- qtlGrp[qtlGrp$string==qtlGrp$string[i],]

for(j in 1:dim(envcomb)[1]){

if (envcomb[j,1]==1){

combgrp[k+j-1,1:ntraits] <- set[1,1:ntraits]

}

if(nsites>1){

if (envcomb[j,2]==1){

combgrp[k+j-1,(ntraits+1):(ntraits*2)] <-

set[1,1:ntraits]

}

}

if(nsites>2){

if (envcomb[j,3]==1){

combgrp[k+j-1,(ntraits*2+1):(ntraits*3)] <-

set[1,1:ntraits]

}

}

if (nsites==2){

if (envcomb$sum[j]==1)combgrp$N[k+j-1] <-

ceiling(set$N[1]*nonoverlapped)

if (envcomb$sum[j]==2)combgrp$N[k+j-1] <-

ceiling(set$N[1]*overlapped)

}

if(nsites==3){

if (envcomb$sum[j]==1)combgrp$N[k+j-1] <-

ceiling(set$N[1]*nonoverlapped*nonoverlapped)

if (envcomb$sum[j]==2)combgrp$N[k+j-1] <-

ceiling(set$N[1]*overlapped*nonoverlapped)

if (envcomb$sum[j]==3)combgrp$N[k+j-1] <-

ceiling(set$N[1]*overlapped*overlapped)

}

}

k <- k + dim(envcomb)[1]

}

combgrp$N <- ceiling(combgrp$N)

total <- sum(combgrp$N)

x <- data.matrix(t(combgrp[,1:(ntraits*nsites)]))

ttol <- data.frame(nqtl=x%*%combgrp$N)

return (list(combgrp=combgrp,trait_total=ttol,

ngrps=dim(combgrp)[1],total=total))

}

#***************************************************************

# Sampling for qtl effects from a normal distribution

# Generate effects with given covariance matrix

#***************************************************************

effects_sampling <- function(grp,gl,nqtl,ntraits,seed,tname){

grp <- grp[grp$N>0,]

y <- as.data.frame(matrix(0,nqtl,ntraits))

set.seed(seed);

for (i in 1:(ntraits)){

y[,i] <- rnorm(nqtl,0,1)

}

names(y) <- tname

ph <- as.matrix(y)%*%gl

ph <- data.frame(ph)

names(ph) <- names(y)

#***************************************************************

# Assign qtl effects according the sharing groups

#***************************************************************

rand <- as.data.frame(matrix(0,nqtl,ntraits+1))

names(rand) <- c('qtl',paste('p',1:ntraits,sep=''))

rand$qtl <- seq(1:nqtl)

rand[,2:(ntraits+1)] <- 1

rand$rand <- runif(nqtl)

kstart=0

for (idx in 1:dim(grp)[1]){

if (idx==1) {

kstart <- 1

} else {

kstart <- sum(grp$N[1:(idx-1)])+1

}

kend <- sum(grp$N[1:idx])

for (i in 1:ntraits){

if (grp[idx,i]==0)

rand[kstart:kend,i+1] <- 0

}

}

rand <- rand[order(rand$rand),]

rand$qtl <- seq(1:nqtl)

rand <- rand[,2:(ntraits+1)]

effect <- ph

for (i in 1:ntraits){

effect[,i] <- ifelse(rand[,i]==1,effect[,i],0)

}

correl <- cor(effect)

rand$sum <- rowMeans(rand[,c(1:ntraits)])*ntraits

effect$qtl <- seq(1:dim(effect)[1])

effect <- effect[,c(dim(effect)[2],1:(ntraits))]

effect <- cbind(effect,rand)

effect <- effect[effect$sum!=0,]

effect$qtl <- seq(1:dim(effect)[1])

pos<- data.frame(snp=seq(1:nrow(effect)),rand=runif(nrow(effect)))

pos <- pos[order(pos$rand),]

effect$pos <- pos$snp[1:dim(effect)[1]]

return (effect)

}

#***************************************************************

# RescaleQTL effects to variance of 1 and mean of 0

#***************************************************************

effects_rescaling <- function(d,ntraits,nsites) {

for (j in 1:(ntraits*nsites)){

d <- d[order(-d[,j+(ntraits*nsites)]),]

v=d[d[,j+(ntraits*nsites)]==1,]

xbar <- mean(v[,j])

std <- sqrt(var(v[,j]))

d[,j]=ifelse(d[,j+(ntraits*nsites)]==1,(d[,j]-xbar)/std,NA)

v=d[d[,j+(ntraits*nsites)]==1,]

xbar <- mean(v[,j])

}

return (d)

}

#***************************************************************

# MAIN PROGRAM

#***************************************************************

#***************************************************************

# Input parameters

#***************************************************************

# Traitnames to simulate

traits <- c('dis','yld')

# Environment names to simulate

sites <- c('A','B')

# Percentage of QTL overlapped between traits

# within the same environment

traitoverlap <- 0.2

# Percentage of QTL overlapped between environments

# for the same trait

envoverlap <- 0.7

# Random seed for generating pseudo random numbers

seed <- 12345

# Number of QTL controlling one trait

numqtl <- 1000

# Minimum minor allele frequency for a SNP used as QTL

mafmin <- 0.1

# Total number of SNPs on the genome used for simulation

nmarkers <- 5000

# Correlation matrix between QTL for an example

# with two traits across two environments

corInput <- as.matrix(read.table(textConnection(

'1.0 0.3 0.8 0.0

0.3 1.0 0.0 0.4

0.8 0.0 1.0 0.3

0.0 0.4 0.3 1.0'

),header=F,sep=''))

traitnames <- c('dis_A','yld_A','dis_B','yld_B')

colnames(corInput) <- traitnames

rownames(corInput) <- traitnames

ntraits <- length(traits)

nsites <- length(sites)

nt <- ntraits*nsites

if (ntraits>4){

stop(paste0('Number of traits are too many.',

'The package cannot simulate QTL for more than ',

'4 traits.\n Program stopped.\n'))

}

if (nsites>3){

stop(paste0('Number of environments are too many.',

'The package cannot simulate QTL for more than ',

'3 environments.\nProgram stopped.\n'))

}

if (nrow(corInput)!=ncol(corInput)){

stop(paste0('Numbers of rows or columns of the correlation ',

'matrix are not equal to trait and environment ',

'combinations.\n Please check the rank of the ',

'correlation matrix...\n'))

}

if (nrow(corInput)!=ncol(corInput) &

nrow(corInput)!=length(traitnames)){

stop('Correlation matrix does not have ')

}

cat('\n\nQTL simulation for',ntraits,'traits at',nsites,

'environments:\n\n')

for (i in 1:ntraits){

cat('trait',i,': ',traits[i],'\n')

}

cat('\n')

for (i in 1:nsites){

cat('Environment',i,': ',sites[i],'\n')

}

cat('\n')

cat('QTL overlapping between traits: ',

traitoverlap,'\n\n')

cat('QTL overlapping between environments: ',

envoverlap,'\n\n')

cat('Random seed used: ',

seed,'\n\n')

cat('Targeted correlations between the effects of traits:\n\n')

print(corInput)

cat('\n')

#***************************************************************

# Cholesky decomposition of the correlation matrix

#***************************************************************

gl <- chol(corInput)

#***************************************************************

# Arrange sharing groups between traits and environments

#***************************************************************

groups <- group_combining(ntraits=ntraits,

nsites=nsites,

nqtl=numqtl,

traitoverlap=traitoverlap,

envoverlap=envoverlap)

# Total number QTL simulated

nqtl_total <- groups$total

# Sharing groups between traits and environments

qtlgrp <- groups$combgrp

qtlgrp <- qtlgrp[qtlgrp$N>0,]

# Number of QTL per trait

nqtlp <- groups$trait_total

row.names(nqtlp) <- traitnames

cat('Total number of QTL simulated: ',

nqtl_total,'\n\n')

cat('Number of QTL per trait-environment: \n')

print(nqtlp)

#***************************************************************

# Sampling effects for the QTL

#***************************************************************

addqtl <- effects_sampling(grp=qtlgrp,

gl=as.matrix(chol(corInput)),

nqtl=nqtl_total,

ntraits=ntraits*nsites,

seed=seed,

tname=traitnames

)

#***************************************************************

# Assign QTL randomly along whole genome

#

# qtlout$pos shows the position of QTL on the genome

#***************************************************************

qtlout=addqtl[,c(-1)]

pos<- data.frame(snp=seq(1:nmarkers),rand=runif(nmarkers))

pos <- pos[order(pos$rand),]

qtlout$pos <- pos$snp[1:dim(qtlout)[1]]

#***************************************************************

# QTL effect rescaling within trait (variance=1 and mean=0)

#***************************************************************

qtleffects <- effects_rescaling (d=qtlout,

ntraits=ntraits,

nsites=nsites)

qtleffects <- qtleffects[order(qtleffects$pos),]

qtleffects <- qtleffects[,names(qtleffects) %in%

c(traitnames,'pos')]

head(qtleffects)

#***************************************************************

# Calculating correlations between simulated QTL

#***************************************************************

cor(qtleffects[,1:4],use = 'complete')
